# Material Engineering Solutions toward Selective Redox Catalysts for Chemical-Looping-Based Olefin Production Schemes: A Review

**DOI:** 10.1021/acs.energyfuels.4c03196

**Published:** 2024-09-10

**Authors:** Alexander Oing, Elena von Müller, Felix Donat, Christoph R. Müller

**Affiliations:** Laboratory of Energy Science and Engineering, Department of Mechanical and Process Engineering, ETH Zurich, Leonhardstrasse 21, 8092 Zürich, Switzerland

## Abstract

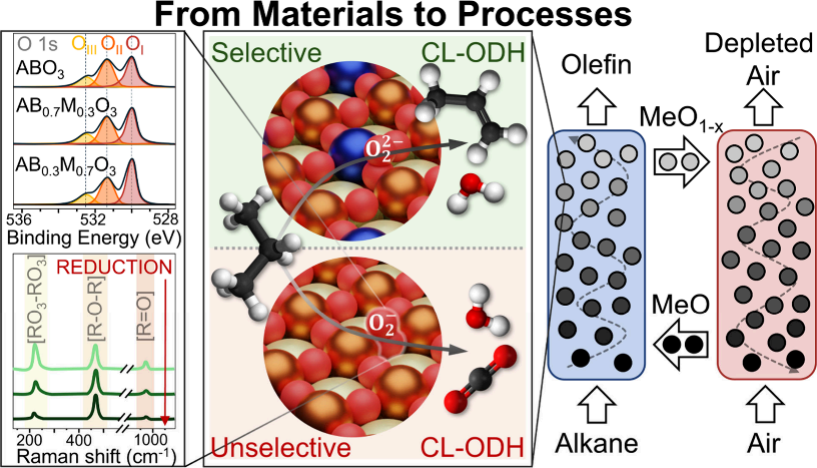

Chemical looping (CL) has emerged as a promising approach in the oxidative dehydrogenation (ODH) of light alkanes, offering an opportunity for significant reductions in emissions and energy consumption in the ethylene and propylene production industry. While high olefin yields are achievable via CL, the material requirements (e.g., electronic and geometric structures) that prevent the total conversion of alkanes to CO_*x*_ are not clearly understood. This review aims to give a concise understanding of the nucleophilic oxygen species involved in the selective reaction pathways for olefin production as well as of the electrophilic oxygen species that promote an overoxidation to CO_*x*_ products. It further introduces advanced characterization techniques such as X-ray photoelectron spectroscopy, Raman spectroscopy, electron paramagnetic resonance spectroscopy, and resonant inelastic X-ray scattering, which have been employed successfully in identifying such reactive oxygen species. To mitigate CO_*x*_ formation and enhance olefin selectivity, material engineering solutions are discussed. Common techniques include doping of the bulk or surface and the deposition of functional coatings. In the context of energy consumption and CO_2_ intensity, techno-economic assessments of CL-ODH systems have predicted energy savings of up to 80% compared to established olefin production processes such as steam cracking or dehydrogenation. Finally, although their practical application has been limited to date, the potential advantages of the use of fluidized bed reactors in CL-ODH are presented.

## 1 Introduction

Light olefins such as ethylene, propylene, or butylene rank among the most important bulk products in the chemical industry today. In fact, ethylene and propylene place first and second, respectively, among the organic chemical compounds produced worldwide.^1^ The applications of light olefins are vast, ranging from their polymerization to yield plastics and synthetic rubbers to their usage as precursors for the synthesis of other platform and fine chemicals.^2,3^ Light olefins are key building blocks for consumer goods in everyday life, and their global production is currently increasing by 3–4% annually, resulting in a predicted global production estimate of over 400 Mt of ethylene and propylene in 2030.^4^

Currently, light olefins are produced largely through the steam and fluid catalytic cracking of naphtha and gaseous hydrocarbons.^5^ These well-established processes have been improved steadily over decades, leading to thermal efficiencies of up to 95% and hence very little room for further optimization.^6^ Despite their high thermal efficiencies, cracking processes are very energy intensive, owing to the endothermicity of the cracking reactions (Δ*H*^R^_777 K_ = 70–100 kJ/mol).^7^ As a result, ethylene production alone is reported to account for 15% of the total energy consumption of the chemical industry.^4^ Furthermore, the heat required to drive the endothermic reaction is generated by the combustion of hydrocarbons in the cracking furnace, resulting in a considerable CO_2_ production (over 300 million tons of CO_2_/year).^8,9^ Consequently, the contribution of the olefin production to the global CO_2_ emissions is estimated to be about 1.7%.^4^ Considering the prevailing challenge to mitigate climate change by reducing anthropogenic CO_2_ emissions, there is a pressing need for the development of more sustainable technologies for olefin production to meet the predicted global rise in their demand, while simultaneously decreasing the carbon footprint of the industry.

The shale gas revolution in the United States has led to a significant increase in the availability of light alkanes at low cost, which has promoted dehydrogenation processes as an alternative route for olefin production.^2^ In particular for propylene production, propane dehydrogenation has become an economically established alternative, e.g., through the Catofin and Oleflex processes which currently account for about 10% of the global propylene production.^9,10^ An advantage of the dehydrogenation of light alkanes over cracking processes is a higher product selectivity (up to 88%),^9^ which substantially reduces costly downstream product separation. Nevertheless, thermodynamic limitations constrain the overall productivity of dehydrogenation, and the overall energy penalty remains relatively large despite the lower reaction temperatures (generally between 500 and 600 °C) when compared to the cracking processes (generally between 800 and 900 °C),^8^ as the dehydrogenation reaction (eq 1.1) is even more endothermic (Δ*H*^R^_823 K_ = 130–143 kJ/mol).^9,11,12^ A promising alternative reaction pathway to circumvent the endothermicity of light olefin production is the oxidative dehydrogenation (ODH) of light alkanes. In ODH, an alkane reacts with oxygen to form the respective olefin and water as a byproduct (instead of hydrogen), thus rendering the overall reaction exothermic (Δ*H*^R^_823 K_ = −116 to −103 kJ/mol, depending on the alkane)^9,12^ (eq 1.2).
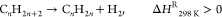
1.1
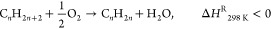
1.2

While it can be argued that burning the H_2_ produced from alkane dehydrogenation or cracking could offset the hypothetical advantage of energy savings due to the exothermic ODH reaction, ODH processes have the additional benefit of a higher olefin productivity, as they shift the thermodynamic equilibrium toward the product side compared to (nonoxidative) dehydrogenation (Δ*G*^R^_dehydrogenation,298 K_ = 86–101 kJ/mol and Δ*G*^R^_ODH,298 K_ = −142 to −128 kJ/mol).

In the conventional ODH reaction scheme, gaseous oxygen is cofed with alkanes to produce olefins, which not only poses a potential safety hazard but also necessitates costly air separation processes for oxygen generation. To address these issues, it has been proposed to integrate the ODH reaction into a chemical looping (CL) scheme. In CL, a chemical intermediate, usually a solid metal oxide (often termed “oxygen carrier” or “redox catalyst”), facilitates the splitting of the desired reaction into two or more spatially or temporally separated subreactions, creating a closed redox loop. Initially developed for the combustion of fuels in the absence of air to produce highly concentrated CO_2_ streams for storage or conversion,^13,14^ CL has recently been investigated to be applied to various fields in chemical production, such as CO_2_ and water splitting, air separation, reforming, and ODH.^15−17^ Prof. Adanez and his co-workers have pioneered the CL research area through seminal studies on the development and design of efficient oxygen carriers^18−20^ and their use in an energy-related context,^21−24^ and thus formed the basis for our current understanding of their functioning under industrially relevant conditions. The field has since extended rapidly exploiting multiple conceptual advantages of CL over conventional hydrocarbon conversion schemes,^25,26^ with oxygen carriers also encompassing catalytic properties, hence the term “redox catalyst”. In CL-ODH, the redox catalyst serves as an oxygen donor in the first half-cycle of the redox loop by supplying its lattice oxygen to the ODH reaction. Subsequently, the redox catalyst is regenerated in air (or other oxidants) during the subsequent half-cycle. In this manner, the necessity of costly air separation for the ODH reaction is circumvented and the cofeeding of gaseous oxygen into the reactor is avoided. Implementing a CL scheme into ODH also presents benefits for process optimization through heat integration since both half-cycles, the ODH reaction and the reoxidation reaction, can be tailored to be net exothermic.^26−28^ It is even possible to integrate CO_2_ valorization into CL-ODH, as some redox catalysts may be reoxidized by CO_2_ that is reduced to CO.^29^

**Figure 1:**
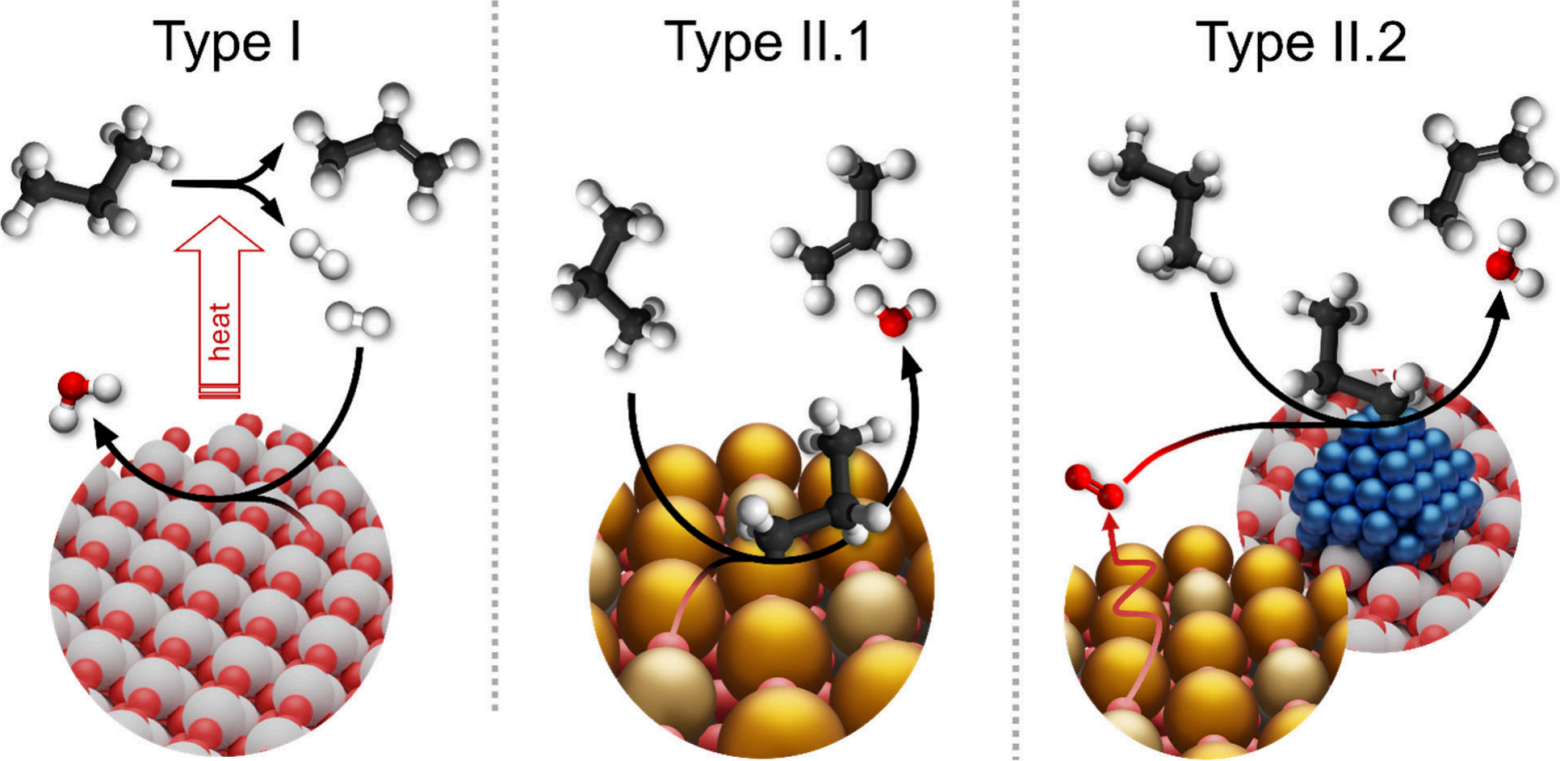
Schematic illustration of the different CL-ODH schemes. Type I: The redox catalyst selectively combusts hydrogen to provide heat for the olefin production through gas phase dehydrogenation. Type II.1: The redox catalyst is a dual functional material that catalyzes the ODH reaction and donates lattice oxygen to the reaction. Type II.2: A tandem catalyst of materials with split functionality is employed that, e.g., combines a metal oxide releasing gaseous oxygen with an ODH catalyst.

In general, there are two different approaches to olefin production via CL (Figure 1). In the first approach (type I), alkane dehydrogenation occurs at high temperatures through gas phase dehydrogenation primarily. The hydrogen that is produced is then selectively combusted by the redox catalyst, supplying also heat for the endothermic gas phase dehydrogenation reaction.^30−34^ Most type I redox catalysts are operated at temperatures above 650 °C; however, there exists no clear dividing temperature for the different types of redox catalysts, as the onset of thermal decomposition depends on various factors, such as the type of alkane (ethane, propane, or butane), the space velocity, and the reactor design. It is therefore essential to carry out control experiments with an empty reactor to distinguish between the contribution of the redox catalyst and thermal gas phase decomposition or interactions with the reactor material. The second approach (type II) includes a heterogeneous reaction between the gaseous alkanes and the surface of the redox catalyst to produce olefins via, e.g., a Mars–van Krevelen (MvK) mechanism.^26^ Type II CL-ODH reactions are generally conducted at temperatures below 650 °C and can be divided further into two subcategories: In a type II.1 CL-ODH reaction, the redox catalyst has a dual functionality by simultaneously catalyzing the dehydrogenation reaction and donating its lattice oxygen.^35−44^ Type II.2 CL-ODH schemes use a combination of materials, and the functionality is split between the individual materials that are involved in the reaction; i.e., one material catalyzes the dehydrogenation reaction and another material supplies oxygen to the reaction. These tandem catalytic systems can be realized either by combining a dehydrogenation catalyst with a redox catalyst that selectively combusts hydrogen or by pairing an ODH catalyst with a metal oxide capable of releasing gaseous oxygen under reaction conditions.^45−48^ It is also noteworthy that, although the differentiation between type I and type II redox catalysts is based on their primary olefin production pathway, it is in fact possible for both reaction pathways to occur over one type of redox catalyst. Concerning the desirable properties of a redox catalyst, it should possess a high activity and product selectivity, as well as a high cycling stability and oxygen storage capacity (e.g., OSC > 1 wt %), to produce olefins economically.^26,49^

**Figure 2:**
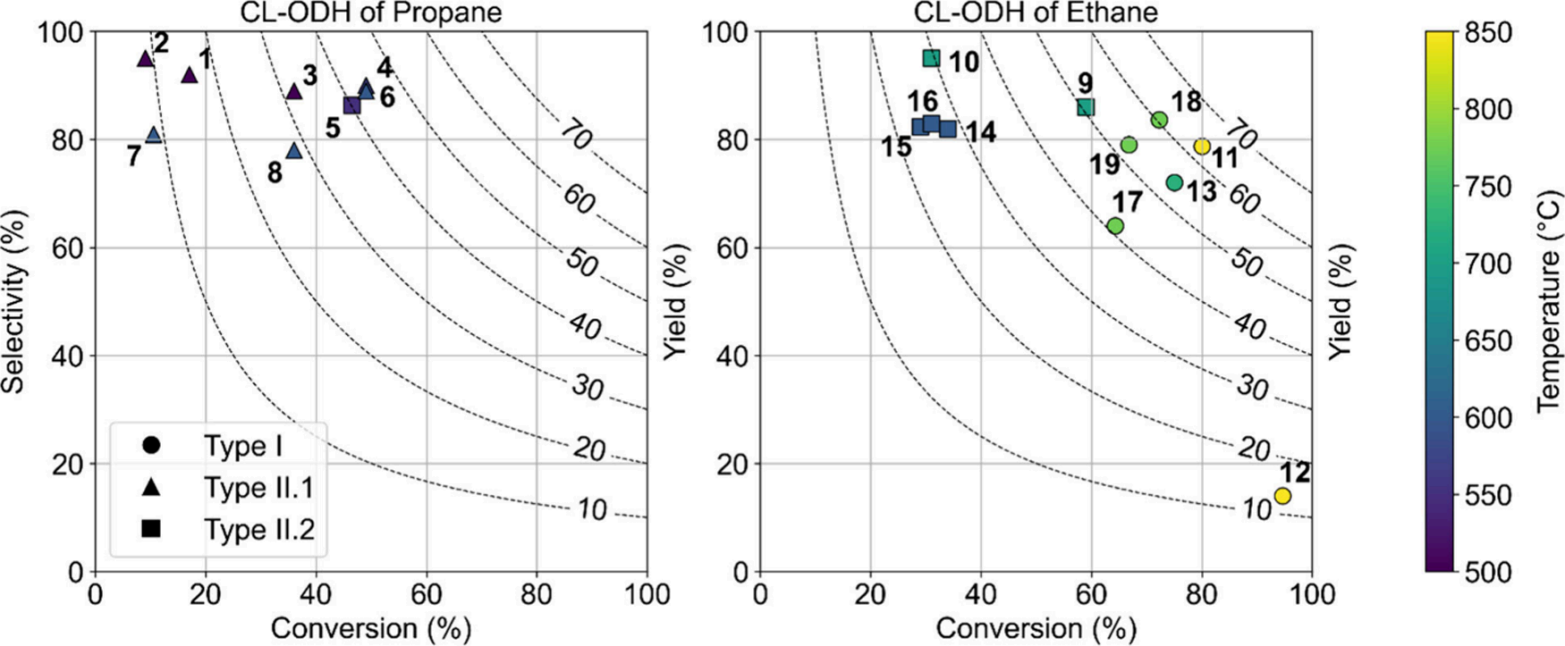
Overview of the performance of selected redox catalysts for the CL-ODH of propane (left) and ethane (right). 1, 1VO_*x*_–TiO_2_;^51^ 2, 0.1VO_*x*_–TiO_2_;^51^ 3, (Mo/V)O_*x*_;^52^ 4, Fe_2_O_3_@MoO_3_;^49^ 5, FeVO_4_–VO_*x*_;^46^ 6, VO_*x*_/CeO_2_–Al_2_O_3_;^53^ 7, CeO_2_–Al_2_O_3_;^53^ 8, VO_*x*_–Al_2_O_3_;^53^ 9, La_0.8_Sr_.0.2_FeO_3_@Li_2_CO_3_;^54^ 10, LaFeO_3_@Li_2_CO_3_;^54^ 11, Mg_6_MnO_8_@Na_2_WO_4_;^31^ 12, Mg_6_MnO_8_;^31^ 13, CaTi_0.1_Mn_.0.9_O_3_@Na_2_MoO_4_;^55^ 14, Sr_0.8_Ca_.0.2_FeO_3_–V_2_O_5_;^47^ 15, Sr_0.8_Ca_.0.2_FeO_3_;^47^ 16, V_2_O_5_;^47^ 17, LaMnO_3_;^56^ 18, LaMnO_3_@Na_2_WO_4_;^56^ 19, LaMnO_3_@Na_3_PO_4_.^56^ Specific reactor conditions are tabulated in Table 1.

A performance overview of selected catalysts in the ODH of ethane and propane is provided in Figure 2, while details on the applied experimental conditions are summarized in Table 1. The reported catalytic performance parameters of the catalysts should be considered with respect to the specific reaction conditions, for instance the amount of catalyst used (*m*_cat_) or the volumetric flow rate of gas through the catalytic bed, i.e., the gas hourly space velocity (GHSV). The comparison of standardized performance metrics such as the turnover frequency (TOF, a measure of the catalytic activity per active site) or the space-time yield (STY, a measure of the amount of product formed per unit mass of catalyst and time) is more suited for judging the intrinsic activity of catalysts and their industrial performance.

**Table 1:** Comparison of Reactor Test Conditions of Selected CL-ODH Catalysts (Ethane, ODHE; Propane, ODHP) Presented in Figure 2

no.	catalyst	reaction	conv (%)	sel (%)	temp (°C)	ratio^a^ (vol %)	*m*_cat_ (g)	GHSV (s^–1^)	flow rate (mL/min)
1	1VO_*x*_–TiO_2_^51^	ODHP	17	92	500	19:81 (N_2_)	0.5	2500	21
2	0.1VO_*x*_–TiO_2_^51^	ODHP	9	95	500	19:81 (N_2_)	0.5	2500	21
3	(Mo/V)O_*x*_^52^	ODHP	36	89	500	19:81 (N_2_)	0.5	2500	21
4	Fe_2_O_3_@MoO_3_^49^	ODHP	49	90	570	19:81 (N_2_)	0.5	3000	21
5	FeVO_4_–VO^46^	ODHP	47	86	550	10:90 (N_2_)	0.5	2500	22
6	VO_*x*_/CeO_2_–Al_2_O_3_^53^	ODHP	49	89	600	n.a.	0.5	2500	20
7	CeO_2_–Al_2_O_3_^53^	ODHP	11	81	600	n.a.	0.5	2500	20
8	VO_*x*_–Al_2_O_3_^53^	ODHP	36	78	600	n.a.	0.5	2500	20
9	La_0.8_Sr_.0.2_FeO_3_@Li_2_CO_3_^54^	ODHE	59	86	700	n.a. (Ar)	5	480	20–80
10	LaFeO_3_@Li_2_CO_3_^54^	ODHE	31	95	700	n.a. (Ar)	5	480	20–80
11	Mg_6_MnO_8_@Na_2_WO_4_^57^	ODHE	80	79	850	80:20 (Ar)	5	4500	5.25
12	Mg_6_MnO_8_^57^	ODHE	94.6	14	850	80:20 (Ar)	5	4500	5.25
13	CaTi_0.1_Mn_.0.9_O_3_@Na_2_MoO_4_^27^	ODHE	75	72	725	80:20 (N_2_)	n.a.	75	n.a.
14	Sr_0.8_Ca_.0.2_FeO_3_ + V_2_O_5_/SiO_2_^47^	ODHE	34	82	600	14:86 (N_2_)	10	6000	40
15	Sr_0.8_Ca_.0.2_FeO_3_/SiO_2_^47^	ODHE	29	82	600	14:86 (N_2_)	10	6000	40
16	V_2_O_5_/SiO_2_^47^	ODHE	31	83	600	14:86 (N_2_)	10	6000	40
17	LaMnO_3_^56^	ODHE	64	64	775	40:60 (He)	2	3400	n.a.
18	LaMnO_3_@Na_2_WO_4_^56^	ODHE	72	84	775	40:60 (He)	2	3400	n.a.
19	LaMnO_3_@Na_3_PO_4_^56^	ODHE	67	79	775	40:60 (He)	2	3400	n.a.

a Ratio of alkane (propane/ethane) to diluting gas. The nature of the dilutant is specified in parentheses.

Despite its numerous theoretical advantages, CL-ODH has not yet been demonstrated at industrially relevant scales, which is largely because redox catalysts tend to overoxidize alkanes (and the olefin products) to CO_*x*_. The total oxidation of the hydrocarbons by redox catalysts has been linked to electrophilic oxygen species, while nucleophilic oxygen species have been associated with the selective production of olefins.^50^

This review aims to provide a perspective on current material engineering solutions to increase the selectivity of redox catalyst in CL processes for olefin production. First, the current understanding of the origin of overoxidation of redox catalysts is summarized. This is followed by an overview of different material engineering approaches to create highly selective redox catalysts and circumvent their tendency toward total oxidation. Finally, a brief outlook is given on the potential energy and CO_2_ emission savings of CL-ODH processes compared to conventional olefin production, as well as a short stance on the viability of implementing redox catalysts in industrial olefin production plants. This review article bridges the most recent advances in CL-ODH across scales, from addressing the characterization of unselective oxygen species at an atomic level to presenting the overall benefits of CL-ODH on a process scale. Consequently, this review may serve as a guideline for implementing the described characterization techniques to improve the understanding of the mechanisms that control overoxidation, progress the development of selective redox catalysts, and ultimately advance the technology toward industrial implementation.

## 2 The Role of Oxygen Species in Overoxidation

### 2.1 Overoxidation Mechanisms

The ODH reaction of alkanes to olefins generally follows the MvK mechanism. Initially, alkanes adsorb onto the catalytic sites at the surface of the redox catalyst. There, the C–H bond of alkanes is activated by nearby metallic species, while hydrogen is abstracted by nearby oxygen species, following eq 2.1:^58^
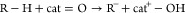
2.1

The alkyl radical R^–^ can undergo further reactions, depending on the chemical potential of the oxygen species involved. An unfavorable pathway is to undergo total oxidation, forming stable total oxidation products such as CO_2_ or CO. Alternatively, the alkyl radical can undergo a reaction pathway to form the desired olefin C_*n*_H_2*n*_. It should, however, be noted that olefins are highly reactive and can readsorb to the catalyst surface, making them prone to further conversion into total oxidation products.

Addressing the mechanism(s) of hydrocarbon oxidation, surface oxide O^2–^ and peroxide O_2_^2–^ ions have been identified as nucleophilic species^50,58^ and have been linked to the selective oxidation of alkanes to olefins, without their overoxidation to CO_*x*_. These nucleophilic species are protonated during the C–H activation process to form −OH (eq 2.1). The alkyl radicals undergo C=C bond formation yielding olefins, while the abstracted hydrogen species are ultimately released in the form of H_2_O, resulting in the formation of an oxygen vacancy. The resultant (surface) oxygen vacancy yields a gradient in oxygen concentration across the oxide lattice, which is compensated for by the migration of ionic oxygen species from the bulk to the surface. Electrophilic oxygen species, such as O^–^ and superoxide O_2_^–^, tend to interact with bonds of higher electron density in the hydrocarbon, viz. the π-bonds of olefins.^50^ In this scenario, the formed olefin species are overoxidized, yielding CO_*x*_.

To elucidate on why it is the electrophilic rather than nucleophilic oxygen species that cause the total oxidation of hydrocarbons, one can take the ODH of ethane as an example. After the successful conversion of C_2_H_6_, the formed ethylene is adsorbed onto the surface (adsorption energy, *E*_a_). There exist subsequent reaction pathways which do not involve oxygen species at all, e.g., the desorption of the produced C_2_H_4_, or its cracking yielding CH_2_* species. Considering the scenarios which do involve an interaction with oxygen species, further hydrogen abstraction from C_2_H_4_ yielding C_2_H_3_* may occur. This pathway requires an energy input *E*_n_ and is initiated by nucleophilic oxygen species. If *E*_n_ > *E*_a_, this scenario is energetically unfavorable to occur. Alternatively, an interaction with electrophilic oxygen species leads to the formation of HO* and C_2_H_3_O*, which may further dissociate into CO_*x*_ species.^59^

### 2.2 Characterization Techniques to Uncover the Nature of Oxygen Species

**Figure 3:**
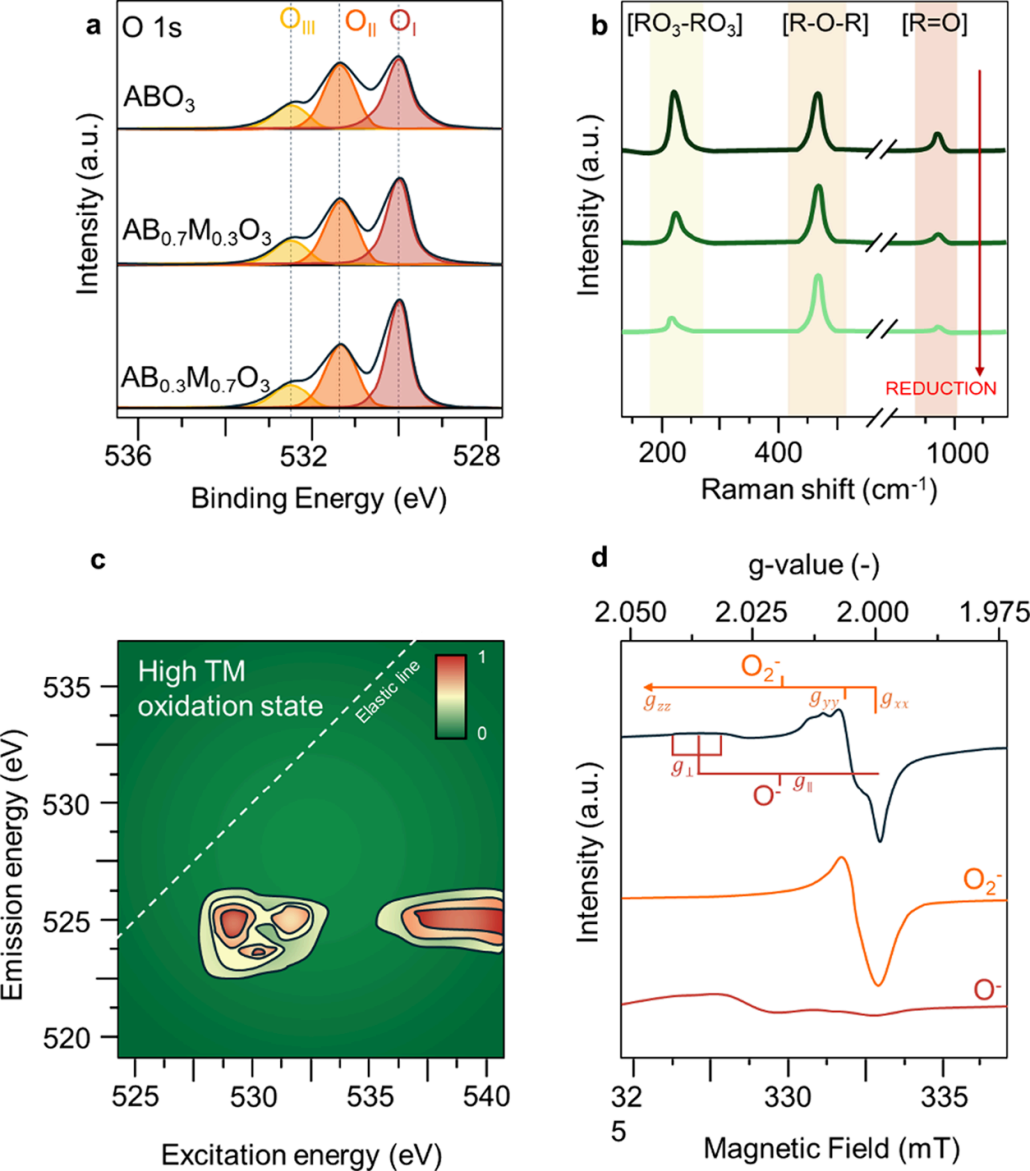
Overview of different experimental techniques to identify the various oxygen species that can be present in metal oxides. Note that the figures do not show real data but rather aim to guide the reader in the interpretation of qualitative changes in features of redox catalysts in chemical looping. (a) Schematic O 1s core-level XPS of a perovskite AB_1–*x*_M_*x*_O_3_ (redox catalyst) deconvoluted into three contributions: O_I_ (lattice oxygen), O_II_ (electrophilic oxygen species), and O_III_ (hydroxide or carbonate species). (b) Schematic in situ Raman spectra of a redox catalyst during reduction. (c) Schematic O K-edge RIXS map of a TM oxide featuring TM-O hybridization features. (d) Schematic powder EPR spectra in the oxygen radical fingerprint region with contributions of O_2_^–^ and O^–^ anions.

Various oxygen species are involved during the reduction of a redox catalyst, with ionic oxygen species migrating from the surface to the bulk and vice versa. Potential oxygen species include O^2–^, O_2_^2–^, O^–^, and O_2_^–^, and such species have been argued to interact with hydrocarbons as nucleophiles or electrophiles as outlined above.^60^ Probing the type of oxygen species being present in the oxygen carrier is a formidable challenge, and techniques used to identify such oxygen species include X-ray photoelectron spectroscopy (XPS), Raman spectroscopy, electron paramagnetic resonance (EPR) spectroscopy, and resonant inelastic X-ray scattering (RIXS), illustrated in Figure 3.

In XPS, the deconvolution of the O 1s signal allows the identification of the different (surface) oxygen species, depending on their binding energies.^61−64^ Often, three main types of oxygen species are considered, viz. lattice oxygen species (O_I_) at ∼528–531 eV, electrophilic oxygen species (O_II_) at ∼531–532 eV, and hydroxide or carbonate species (O_III_) at ∼532–534 eV, as shown in Figure 3a. The exact locations of these peaks depend on the nature and oxidation states of the neighboring metal cations. The assignment of the O_II_ peak at 531–532 eV has been a cause for debate in the literature. Frankcombe et al.^62^ carried out density functional theory (DFT) calculations to evaluate the binding energy of the core electrons and to elucidate the origin of the peak at 531 eV. It was found that the signal may be due to chemisorbed water species (which are more strongly bound to the surface than the species giving rise to the O_III_ peak) or surface hydroxides. The authors also did not reject the possibility that the signal may be due to oxygen in the vicinity of oxygen vacancies. Hence, to probe the evolution of the concentration of oxygen vacancies in a metal oxide, the metal cation should be studied simultaneously by XPS or X-ray absorption near edge spectroscopy (XANES).^65^

Raman spectroscopy has been applied to probe the structure and bonding environment of oxygen species and used in the context of chemical looping to determine the catalytically active centers of redox catalysts. For instance, by monitoring the intensities of the various oxygen bands during the reduction of a redox catalyst, one can deduce the nature of oxygen sites that are involved in the oxidation of hydrocarbons; cf. Figure 3b.^66^

RIXS studies the photon emission energies of excited valence electrons at distinct excitation energies and as such can detect metal–ligand interactions and charge transfer effects.^67^ Electrophilic oxygen O^(2−δ)–^ species, which originate from the presence of hole states on oxygen, appear as a signal at an excitation energy of 531 eV and an emission energy of 523–524 eV; cf. Figure 3c.^68^ RIXS has been applied frequently in electrochemistry to study the oxygen redox activity in Li-ion cathodes.^69^ Distinctive signals at an emission energy of 525 eV can be attributed to transition metal (TM)–O hybridization features and vary in intensity as a function of the TM oxidation state.

EPR spectroscopy is used to probe species with unpaired electrons in response to an externally applied magnetic field under microwave irradiation. It has been widely applied to study oxygen radicals,^70−73^ e.g., adsorbed oxygen anions such as O_2_^–^ and O^–^ species, as seen in Figure 3d. The unpaired electrons in the oxide anions result in an anisotropic EPR signal. The overall shape of the EPR signal is influenced by the nuclear spin of neighboring atoms. The principal values of the *g* tensor of the O_2_^–^ centers depend on the location of the probed species (bulk or surface) and the nature of the metal cations they are coordinated with.^74^ Sobańska et al.^70^ used EPR in ^17^O-enriched isotopic labeling experiments to quantify oxygen exchange rates. The magnetic nucleus of the ^17^O isotope is particularly useful for EPR detection, as the hyperfine coupling with ^17^O leads to line splitting, which makes the attribution and structural characterization more reliable and detailed.

### 2.3 Oxidation State of Metal Oxides

Two principal types of metal oxides can be distinguished.^75^ A “metal–metal oxide” exists in multiple distinct oxidation states, for instance Fe_2_O_3_/Fe_3_O_4_/FeO/Fe. The oxygen content in the material is discrete, and for a certain threshold value of the oxidizing chemical potential of the gas phase the material transitions from one oxidation state to another. On either side of this threshold value, the thermodynamic properties of the material are unaffected by changes to the oxidizing potential of the gas phase.

The second type of metal oxide is the “nonstoichiometric” type, which exists in a large range of oxidation states: upon lattice oxygen removal, vacancies are formed and the crystal lattice distorts. Once these distortions become too large, the material undergoes a change in crystal structure. The oxidizing potential of this type of metal oxide varies continuously with that of the gas phase. Perovskites, of the general form ABO_3−δ_, are a typical example of such nonstoichiometric metal oxides.

For both types of materials, there are multiple consequences arising from the gradual release of oxygen. First, the depletion of the stored oxygen of the redox catalyst impacts its stoichiometry and may trigger a change of its local structure or, if significant enough, its bulk crystal structure. As a result, the geometric (and potentially also the electronic) structure of the surface catalytic sites is altered. Variations in a material’s (crystalline) structure are commonly investigated using in situ X-ray diffraction (XRD)^45,46,54,69,76^ or neutron powder diffraction (NPD) techniques.^77^ Furthermore, changes to the oxygen content of the redox catalyst cause a redistribution of the electron charge across the lattice. This leads to a change in the oxidation state of the TM cations (which can, e.g., be probed by X-ray absorption spectroscopy (XAS)^49,69^) as well as an evolution in ionic oxygen species. Thus, changes in the oxidation states of metal cations and oxygen anions affect the thermodynamic properties of the catalyst, causing variations in its performance in terms of alkane conversion and olefin selectivity. Finally, for a “nonstoichiometric” material, the progressive consumption of oxygen species participating in the ODH reaction creates oxygen vacancies near the catalytic sites. This results in a gradient in the oxygen concentration across the lattice and triggers oxygen migration from the bulk to the surface (oxygen diffusion and surface concentration in themselves are further discussed below). It should however be noted here that as the oxygen reservoirs (in the bulk of the material) become depleted, the rate of oxygen diffusion to the surface decreases, and hence also the oxidizing potential of the redox catalyst decreases.

### 2.4 Concentration of Oxygen Species on the Surface

An important factor to consider for the ODH reaction is the concentration of oxygen species on the surface of a redox catalyst that is available for interaction with an alkane or olefin product. The total oxidation of an alkane to CO_2_ requires considerably more oxygen atoms than its selective dehydrogenation. For example, the complete oxidation of 1 mol of C_3_H_8_ to 3 mol of CO_2_ requires 10 mol of O, while the ODH of 1 mol of C_3_H_8_ to 1 mol of C_3_H_6_ only consumes 1 mol of O (eqs 2.2 and 2.3):^52^
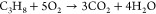
2.2
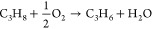
2.3

In the undesirable overoxidation pathway, more moles of oxygen (at a given time) would be removed from the oxygen carrier, leading to a larger quantity of oxygen vacancies per mole of alkane converted and hence a more rapid change in the oxidation state of the metal cations assuming the alkane is converted at the same rate in both pathways (eqs 2.2 and 2.3).

In a kinetic study conducted by Haber et al.,^78^ it was found that prior to surface oxygen species (e.g., O^2–^) being released to the gas phase as diatomic oxygen O_2_, they pass through a series of transient oxygen species, of which some are nucleophilic (O_2_^2–^) and others are electrophilic (O^–^ and O_2_^–^). Aside from depending on the oxygen partial pressure of the gas phase (viz. the driving force for O_2_ release), the rate of change of these transient states is mainly a function of the rate of charge transfer of electrons between oxygen ions and the neighboring metal cations. Thus, Haber et al., accounting for the different exchange rates across species, determined that there exists a mixture of electrophilic and nucleophilic oxygen species at any point in time, whose relative concentrations greatly impact the performance of the ODH redox catalyst. To limit the likelihood of alkane overoxidation, the concentration of electrophilic oxygen species on the surface should be minimized. Zhou et al. conducted DFT calculations of a sulfur-modified NiAl mixed oxide for the ODH of ethane to probe the activity of oxygen species.^59^ It was reported that the sulfate surface modification increased the proportion of Ni^3+^ sites compared to Ni^2+^ sites. Oxygen sites in the vicinity of Ni^3+^ were found to be electrophilic, due to a partial charge transfer from oxygen sites to Ni^3+^ sites, as was shown by Bader charge analysis. It was suggested that, following ethane conversion, ethylene was adsorbed at either metal cation or oxygen sites. When adsorbed on metal cation sites, any further dissociation of the olefin into CO_*x*_ was suggested to be unlikely due to a high energy barrier of 1.30 eV. On the other hand, when adsorbed on oxygen sites, the presence of electrophilic oxygen species after olefin formation was proposed to yield C_2_H_4_(O*)_2_ species. The dissociation of C_2_H_4_(O*)_2_ into CO_*x*_ products was associated with an energy barrier of 0.51 eV, which is lower than the energy barrier for the desorption of ethylene into the gas phase (1.74 eV). This suggested that an overoxidation of ethylene to CO_*x*_, and hence a low olefin yield, is favored in a material containing a high density of electrophilic oxygen species. Minimizing the concentration of electrophilic oxygen species is attempted through material engineering solutions, explored in the following sections.^51^

A notable technique to determine the oxygen surface mobility was developed by Bouwmeester et al.,^79^ which enables calculating the equilibrium surface exchange rates of oxygen. To this end, the redox catalyst is equilibrated in an ^16^O-rich atmosphere for specific conditions of temperature and partial pressure *p*_O_2__. With the use of mass spectrometry (MS), the response to an ^18^O-enriched pulse is measured, by assessing the relative fractions of ^16^O_2_, ^18^O_2_, and ^18^O^16^O present in the outlet gas phase. The overall exchange rate between lattice ^16^O and gaseous ^18^O under equilibrium conditions is evaluated by determining the difference between the fraction of ^18^O at the inlet and that at the outlet of the reactor for a given reactor residence time. Furthermore, the mechanism of the oxygen exchange reaction is proposed to occur in several steps, including the dissociative surface adsorption of diatomic O_2_ from the gas phase and the exchange of dissociated oxygen with lattice oxygen species or another adsorbed O_2_ species. By comparing the relative fractions of oxygen species at the outlet, it is possible to determine which of these steps is rate-limiting.

## 3 Material Solutions to Limit Overoxidation

### 3.1 Type I: Selective Hydrogen Combustion Redox Catalysts

Type I classified materials solely catalyze the combustion of hydrogen and do not participate in the activation of the hydrocarbon. These reactions generally take place at temperatures exceeding 650 °C. In this CL-ODH scheme, alkanes are separately converted into olefins and hydrogen in the gas phase, where hydrogen reacts with the lattice oxygen of a redox catalyst to form H_2_O. Several such redox catalysts with high thermal stabilities and high olefin yields have been developed and are discussed in this section.

Early studies by the group of Rothenberg investigated ceria-based doped oxide materials for the selective combustion of hydrogen which had high redox stabilities, a substantial mass of redox-active lattice oxygen available for reaction (up to ∼2 wt %), and high hydrogen combustion selectivities (up to 93%).^80,81^ The catalytic performance was improved further by lead-containing materials such as PbCrO_4_,^82,83^ maintaining a redox stability for up to 120 redox cycles, containing up to 4.5 wt % redox-active lattice oxygen and achieving nearly 100% hydrogen combustion selectivity. XRD measurements showed a phase transformation to Pb_2_(CrO_4_)O at temperatures exceeding 500 °C. TPR and TPO measurements revealed an irreversibility of the redox cycle for a reduction period longer than 3 min, after which the Pb_2_(CrO_4_)O phase could no longer be recovered upon reoxidation. Hence, PbCrO_4_ is an efficient material for the selective combustion of hydrogen, provided that its redox cycling is performed under reversible conditions.

More recently, Yusuf et al. discussed the advantages of Mg_6_MnO_8_ containing a molten salt surface modification (Na_2_WO_4_, melting point ∼684 °C) as a redox catalyst for the CL-ODH of ethane.^31^ XPS and low-energy ion scattering (LEIS) measurements showed that the modified catalyst was surface-rich in Na and W (and possibly Mg), while the surface of the unmodified material was composed primarily of Mg and Mn. Catalytic tests were carried out at 850 °C to compare the ethane conversion, *X*, and ethylene yield, *Y*, under thermal cracking conditions (empty reactor, *X*_blank_ = 63%, *Y*_blank_ = 57%), Mg_6_MnO_8_ (*X*_plain_ = 94%, *Y*_plain_ = 13%), and Na_2_WO_4_-modified Mg_6_MnO_8_ (*X*_prom_ = 78–83%, *Y*_prom_ = 59–63%). To demonstrate the effectiveness of the molten salt surface modification, CO combustion experiments, with cofeeding of gaseous O_2_, were carried out at 600–800 °C, revealing a nearly 100% CO conversion for the unmodified redox catalyst over the entire temperature range, while the CO conversion of the modified redox catalyst (i.e., containing a coating of Na_2_WO_4_) was as low as 4% at 600 °C, 11% at 700 °C, and 20% at 800 °C. Accordingly, the onset of hydrogen combustion in a mixture of H_2_/C_2_H_4_ was increased from ∼550 °C for Mg_6_MnO_8_ to ∼725 °C for the Na_2_WO_4_-modified Mg_6_MnO_8_. The ability of oxygen to migrate from the bulk Mg_6_MnO_8_ through the Na_2_WO_4_ shell was confirmed by surface oxygen ^18^O_2_–^16^O_2_ exchange experiments in response to pulses of ^18^O_2_ and H_2_. Further, H_2_/O_2_ gas switching experiments revealed a negligible solubility of H_2_ in the Na_2_WO_4_ coating, allowing the authors to conclude that hydrogen combustion of the promoted material occurred at the Na_2_WO_4_/gas interface. The high ethylene yield of 63% was explained by the blocking of sites that favor CO_*x*_ production (possibly Mg and Mn).

To summarize, type I redox catalysts can be tailored to maximize their selectivity to the combustion of hydrogen over the oxidation of alkanes/olefins through material engineering solutions such as the doping of metal redox catalysts^80−83^ or the deposition of surface layers (core–shell-type structures).^31^

### 3.2 Type II.1: Dual Functionality Redox Catalysts

#### 3.2.1 Controlling Olefin Selectivity through Active Site Speciation

Dual functionality redox catalysts both catalyze the ODH reaction and supply lattice oxygen. Such redox catalysts were reported, e.g., by Chen et al.,^51^ who investigated the CL-ODH of propane of VO_*x*_ dispersed on a TiO_2_ support. The high dispersion of the vanadia phase was demonstrated by the absence of diffraction peaks for V loadings of <2 wt %, while orthorhombic V_2_O_5_ crystallites were detected for higher loadings. The amount of vanadia deposited was found to affect the speciation of VO_*x*_ on the support: At low loadings (0.25 wt %), vanadia species were found to be mainly isolated VO_4_ sites. Increasing the V loading led to a polymerization of isolated VO_4_ species until a monolayer of VO_*x*_ was formed at around 1 wt %, followed by the formation of crystalline V_2_O_5_ nanoparticles when the loading was further increased. With the use of Raman spectroscopy, it was shown that in systems containing largely isolated VO_4_ sites V=O and V–O–Ti bonds dominated, while the formation of V–O–V bonds (at the expense of V–O–Ti bonds) was observed when the V loading was increased. Catalytic ODH tests showed that the highest propylene selectivity was achieved on catalysts featuring highly dispersed VO_*x*_ sites, while the overall instantaneous propane conversion increased monotonically (from 8 to 20%) with increasing V loading. In in situ diffuse reflectance infrared Fourier transform spectroscopy (DRIFTS) measurements, vibrations due to acetone (*v*_s_(C=O), 1660 cm^–1^) were observed on catalysts containing crystalline V_2_O_5_. Acetone is a key reaction intermediate in the overoxidation of propane and propylene to CO_2_ and indicates the attack of electrophilic oxygen species on propenyl adsorbates. It was therefore deduced that crystalline V_2_O_5_ facilitated the formation of electrophilic oxygen species at the surface of the redox catalyst, which increased the overoxidation of propane to CO_2_. XPS measurements confirmed an increase in the peak area (binding energy of 532 eV) attributed to electrophilic surface oxygen species with the emergence of crystalline V_2_O_5_ from highly dispersed VO_*x*_ at V loadings of >1 wt %.

#### 3.2.2 Controlling Olefin Selectivity through Metal Doping

Doped metal oxides have attracted significant interest in CL due to their tunability toward a particular application. The introduction of dopants (elements substituting existing atoms in the lattice) in redox catalysts has been shown to limit overoxidation of hydrocarbons in ODH reactions, therefore increasing the selectivity toward the desired olefin. For example, Chen et al.^52^ studied the effect of Mo doping (up to 20 mol %) in V_2_O_5_ for the CL-ODH of propane, finding that the propylene selectivity increased from 70 to 89% compared to undoped γ-Al_2_O_3_-supported V_2_O_5_. The introduction of the dopant Mo into the V_2_O_5_ structure of the redox catalyst was confirmed via aberration-corrected HAADF-STEM. The highly dispersed nature of the Mo species was further confirmed by STEM with energy-dispersive spectroscopy (EDS). Raman spectroscopy revealed that, with decreasing the V/Mo ratio from 18 to 4, Mo–V–O bonds gradually appeared at the expense of V–O–Al bonds. The authors noted that the oxidation state of V at the surface was a key determinant of propylene selectivity, and XPS measurements of the reduced samples (hydrogen treatment at 600 °C for 1 h) revealed that doping with Mo resulted in a high abundance of vanadium in the oxidation states V^4+^ and V^3+^ (and a lower abundance of V^5+^ states). Further, DFT calculations indicated that integrating Mo into the V_2_O_5_ lattice induced a higher binding energy of the V–O bonds, as the oxygen vacancy formation energy was increased from 2.19 eV in undoped V_2_O_5_ to 2.85 eV in Mo-doped V_2_O_5_. The higher oxygen vacancy formation energy in Mo-doped V_2_O_5_ was confirmed experimentally by hydrogen-TPR measurements, showing a monotonic increase in the onset temperature of reduction from undoped to 20 mol % Mo-doped V_2_O_5_. Further, as in situ Raman spectroscopy measurements showed a loss in intensity of the V=O signal for samples exposed in a 20% propane/hydrogen atmosphere, the authors argued that oxygen present in V=O is the source of oxygen in ODH. Conversely, the Raman signal intensity due to V–O–Mo bonds was unaffected when samples were exposed to a 20% propane/hydrogen environment. The authors concluded that Mo doping increased the binding strength between O and V, which in turn reduced the overoxidation of propane to CO_*x*_.

In a further attempt to modulate the metal–oxygen binding strength, Wang et al.^49^ introduced high valence dopants (e.g., Mo) into Fe_2_O_3_ supported on Al_2_O_3_. For a molar ratio of Fe/Mo = 9, the catalyst reached a propylene selectivity of 89% at 49% propane conversion compared to 76% propylene selectivity at 14% propane conversion of undoped Fe_2_O_3_ on Al_2_O_3_. HAADF-STEM and extended X-ray absorption fine structure (EXAFS) confirmed the high dispersion of Mo atoms in the Fe_2_O_3_ matrix, showing that Mo cations were isolated rather than clustered.^49^ The doping of Mo into the Fe_2_O_3_ matrix was further confirmed by XRD, with Fe_2_O_3_ reflections shifted to higher diffraction angles upon the substitution of Fe^3+^ cations by smaller Mo^6+^ cations. When the Mo doping concentration was increased, TEM EDX evidenced the formation of Mo clusters for molar ratios of Fe/Mo > 6, and a separate Fe_2_(MoO_4_)_3_ phase was detected by XRD for redox catalysts with a molar ratio of Fe/Mo = 3. The formation of Mo clusters and a separate Fe_2_(MoO_4_)_3_ phase at higher Mo loadings resulted in a decrease in propane conversion and propylene selectivity. NH_3_ temperature programmed desorption (TPD) measurements revealed an increase in acid site density on the surface of Mo-doped Fe_2_O_3_ (Fe/Mo = 9) compared to undoped Fe_2_O_3_, which was attributed to be the reason for the enhanced activity of the Mo-doped Fe_2_O_3_ catalyst. The increase in propylene selectivity on the other hand was again attributed to stronger metal–oxygen bonds induced by the doping of high valence Mo^6+^ cations into the Fe_2_O_3_ lattice. Indeed, hydrogen- and propane-TPR experiments demonstrated a decrease in reducibility of the redox catalyst, as the reduction of Fe_2_O_3_ to Fe_3_O_4_ shifted toward higher temperatures upon doping with Mo. In addition, kinetic experiments conducted in a TGA showed that the introduction of Mo into the Fe_2_O_3_ matrix led to a higher surface oxygen consumption and a smaller bulk oxygen diffusion coefficient, showcasing how doping with Mo can regulate the evolution of oxygen.

To summarize, tailoring metal dispersion and doping metal oxides can be efficient means to increase olefin selectivity. Concerning metal doping, it was shown that introducing metal cations of higher oxidation state than the host lattice (e.g., Mo^6+^ in V_2_O_5_) increases the strength of the M–O bond (where M is the host metal cation), which in turn increases the olefin selectivity.

### 3.3 Type II.2: Tandem Material Systems with Split Functionality

An efficient design strategy to increase the olefin yield in CL-ODH applications is to split the functionalities of catalyzing alkane dehydrogenation and providing oxygen to the reaction between two different materials, e.g., on separate particles. By decoupling the functionalities, the material properties can be tailored individually, i.e., toward a high OSC or a high olefin selectivity, respectively, without directly affecting each other. The strategy can be realized in different ways, e.g., by coupling an ODH catalyst with a metal oxide that possesses the ability to release gaseous oxygen (henceforth referred to as oxygen carrier, OC). Here, it is critical that the OC itself does not overoxidize the alkane reactant or the alkene product, and surface coatings (e.g., alkali metal nitrates or carbonates) have proved to be successful in inhibiting the interaction of gas phase hydrocarbons with the surface of the OC (similar to the type I core–shell redox catalysts).^45^

#### 3.3.1 Nanostructuring of Materials with Split Functionality

Another approach to split functionalities is to combine the dehydrogenation and hydrogen combustion functionalities on a single particle.^46,48^ Specifically, Wang et al.^46^ showed that decreasing the distance of the active sites between FeVO_4_ nanoparticles and well-dispersed VO_*x*_ on an Al_2_O_3_ support enhanced the propylene selectivity of the CL-ODH of propane system at 550 °C from 56% (when the distance was at the millimeter scale) to above 80% (when the distance was at the nanoscale). The distances (proximities) between the different catalytic sites on FeVO_4_ for selective hydrogen combustion and VO_*x*_ for dehydrogenation of propane were achieved by varying the catalyst preparation, using separate particles (0.4–0.8 mm) of the individual materials for the millimeter scale proximity and a wet impregnation approach for the nanoscale proximity of the individual materials on the same particle. Hydrogen-TPR and in situ XRD measurements revealed a decrease in the reduction temperature of the FeVO_4_ nanoparticles for an increasing proximity between FeVO_4_ and VO_*x*_ sites. Consequently, the authors inferred that a hydrogen spillover effect was at play between the two materials at nanoscale proximity, which ultimately resulted in a higher olefin yield. Further, transient gas switches from Ar to diluted propane in an in situ DRIFTS study revealed the emergence of V–OH bands (3650 cm^–1^) as a result of C–H activation on the VO_*x*_ sites, as well as Al–OH bands (3750 and 3690 cm^–1^) that suggested the cleavage of V–O–Al bonds as hydrogen was transferred from V–OH sites to Al–O sites. The emergence of Al–OH bands under propane flow was significantly more pronounced in the absence of FeVO_4_ on the catalysts. It was inferred that, on FeVO_4_-containing catalysts, atomic H preferably migrated from VO_*x*_ sites to adjacent FeVO_4_ for combustion.^46^ A similar redox catalyst design strategy was employed by Wang et al.,^48^ who synthesized a tandem redox catalyst for the CL-ODH of ethane by embedding Ni^2+^ sites into an HY zeolite and incorporating NiO nanoclusters into the pore structure of the zeolite. Ni^2+^ sites in the HY framework provided Lewis acid sites for the selective dehydrogenation of ethane, while the NiO nanoclusters functioned as oxygen reservoirs to selectively combust hydrogen. DFT calculations revealed that the activation barrier on the NiO nanoclusters for ethane dissociation (0.38 eV) was substantially higher than that for hydrogen dissociation (0.13 eV), and that the oxygen vacancy formation energy for NiO nanoclusters confined in the zeolite framework increased over bulk NiO. This was validated through C_2_H_4_- and hydrogen-TPR experiments revealing a decrease in the reducibility of NiO nanoclusters inside the zeolite framework compared to catalysts containing bulk NiO. Hence, the high selectivity for the oxidation of hydrogen over ethane/ethylene was ultimately attributed to an increase in the Ni–O bond strength for NiO nanoclusters confined in the HY framework.^48^

#### 3.3.2 Redox Catalysts with a Core–Shell-Type Architecture

As outlined above, surface coatings have been efficient means to inhibit overoxidation and increase the olefin yield of redox catalysts in CL-ODH. Examples for such coatings include molten salts or carbonates yielding core–shell-type structures.^54^ The function of the coating shell is to prevent the direct contact between the alkane and unselective oxygen species at the surface of the redox catalyst and to provide new catalytic sites. Gao et al.^54^ synthesized Li_2_CO_3_-promoted La_0.8_Sr_0.2_FeO_3_ (LSF) through wet impregnation and calcination at 800 °C. The high temperature treatment led to the melting of Li_2_CO_3_ (melting point 723 °C), yielding an amorphous shell around the crystalline LSF substrate, as confirmed by TEM. Differential scanning calorimetry (DSC) revealed that the Li_2_CO_3_ shell was in a molten state under operating conditions, despite the reaction temperature (700 °C) being below the melting point of Li_2_CO_3_. The lowered melting point was ascribed to a melting point depression typical for nanosized materials such as the Li_2_CO_3_ shell or the possible formation of a eutectic mixture with small amounts of SrCO_3_. Importantly, the core–shell redox catalyst drastically outperformed the unmodified LSF substrate, increasing the ethylene selectivity from <10 to 90% and the overall ethylene yield from <10 to 50% in the temperature range 700–750 °C. C_2_H_6_/O_2_ gas switching experiments showed that ethane was unable to diffuse through the shell material within the short gas–solid contact time. It is worth noting that at 700 °C thermal cracking of ethane takes place, which makes a definite classification of this redox catalyst between type I and type II difficult. Importantly, the thermal conversion of ethane in an empty reactor was determined to be only 7%, which was significantly lower than the 85% ethane conversion that is observed in the presence of the Li_2_CO_3_-promoted LSF, thus demonstrating that the Li_2_CO_3_ shell participates in catalyzing the ODH reaction. In situ XRD measurements and Mössbauer spectroscopy unveiled that unpromoted LSF underwent a deeper reduction to (La/Sr)_2_FeO_4_ (Ruddlesden–Popper phase) and Fe when in contact with ethane under operating conditions compared to the LSF–Li_2_CO_3_ core–shell redox catalyst, which did not reduce to Fe. The authors further investigated the nature of the oxygen species participating in ODH. Electrochemical impedance spectroscopy (EIS) experiments of pure Li_2_CO_3_ showed that the oxygen ionic conductivity was enhanced upon reaching its melting point, while the electronic conductivity remained low. As a result, an electron conduction to counter O^2–^ migration was not possible and the oxygen transport through the molten Li_2_CO_3_ shell was suggested to have taken place in the form of oxidized oxygen species such as peroxide (O_2_^2–^) or superoxide (O_2_^–^), although EIS cannot differentiate between different oxygen anions. Oxygen transport through the molten promoter shell via CO_4_^2–^ and C_2_O_4_^2–^ was ruled out by reference ^13^C nuclear magnetic resonance (NMR) experiments, as the chemical shifts in the recorded spectra excluded the involvement of C-containing intermediate species other than CO_3_^2–^. Catalytic experiments with a CO_2_ cofeed were carried out to examine whether O_2_^2–^ species in fact actively contributed to the CL-ODH of ethane reaction. Lithium peroxide has been reported to readily react with CO_2_ to form Li_2_CO_3_, and peroxide formation is therefore inhibited in the presence of CO_2_. The ethane conversion was indeed significantly reduced from 85 to 25% when 10% of CO_2_ was introduced in the feed gas, which led the authors to conclude that oxygen migration in the molten Li_2_CO_3_ shell took place in the form of O_2_^2–^ species that actively contributed to the CL-ODH of ethane reaction.

Gao et al. studied also molten halide salt coated metal oxides, such as LiBr-coated LSF (melting point of LiBr ∼465 °C), as redox catalysts for the CL-ODH of butane.^84^ The material was synthesized via the wet impregnation of LiBr on commercial LSF, followed by calcination at 500 °C. The core–shell structure of the synthesized material was confirmed directly via STEM–EDS and indirectly via XPS, which showed only weak signals for Fe, Sr, and La compared to Br. The addition of the LiBr shell increased the 1,3-butene selectivity from 2% (LSF without a shell) to 56% (LSF with a LiBr shell). Measurements of the activation energy for CO_2_ formation for LSF with and without a LiBr shell showed no differences below the melting point of LiBr. Above the melting point of LiBr, however, the activation energy for CO_2_ formation increased significantly, suggesting that the molten salt blocked the highly reactive sites that overoxidize butane on the LSF surface. Ab initio molecular dynamics (AIMD) calculations confirmed that the presence of the molten LiBr shell largely inhibited the overoxidation of butane compared to LSF without a LiBr shell. The AIMD calculations also suggested that Br sites in the molten shell play an active role in the ODH reaction by abstracting hydrogen from butane, forming HBr that subsequently reacts with Li_2_O to regenerate LiBr under the formation of water. Similar to carbonate layers, the layer of molten LiBr also reduced the surface exchange rate of oxygen, limiting the rate of reduction of the redox catalyst and the formation of CO_*x*_.

To summarize, a promising approach to develop active and selective type II.2 redox catalysts is tandem catalysts combining dehydrogenation and selective hydrogen combustion functionalities as well as core–shell architectures. Decoupling the functionality to separate sites/particles allows tailoring the structures of sites/materials to a specific subreaction. When the dehydrogenation and selective hydrogen combustion functionalities are combined, the proximity of these two functionalities at the nanoscale can be critical to maximizing the olefin yield. Concerning core–shell-type redox catalysts, the shell allows control of the transport and potentially also the nature of oxygen species participating in the selective oxidative dehydrogenation of alkanes, limiting in turn the overoxidation of alkanes/olefins to CO_*x*_.^31,84^ Providing direct experimental evidence for the presence of different oxygen species on the surface of the molten shells through the techniques illustrated in Figure 3 is, however, not trivial due to the limitations in analysis temperature.

In brief, the objective of designing more efficient ODH redox catalyst is twofold: enhancing alkane conversion by enriching their surfaces with sites active for alkane adsorption and C–H bond activation and increasing olefin selectivity by providing selective oxygen species while limiting overoxidation. Such functionalities can be provided by different material engineering approaches (or a combination of such) including doping and the addition of functional coatings. Table 2 summarizes the presented catalysts, including their method of synthesis, the material engineering solutions to achieve high olefin yields, and potential challenges associated with their scaling to industrial level.

**Table 2:** Overview of the Discussed CL-ODH Redox Catalysts, Summarizing Their Preparation Methods, Key Material Engineering Solutions, and Challenges

catalyst	category	application	preparation methods	material engineering solution to achieve high olefin yield	challenges for industrial implementation of the
PbCrO_4_^82^	type I	H_2_ combustion	commercially acquired	Introduction of Pb increases the activity and stability of the catalyst.	The utilization of Cr entails ecological risks.
Mg_6_MnO_8_@Na_2_WO_4_^57^	type I	ODHE	wetness impregnation	The suppression of unselective surface sites (Mg/Mn) by the addition of Na_2_WO_4_ increases selectivity while enabling oxygen to permeate through.	The introduction of an Na_2_WO_4_ layer reduces the OSC of the redox catalyst. The molten Na_2_WO_4_ layer may lead to difficulties in large scale operation due to reactor corrosion, loss of promoter, and limited possibility of fluidization.
VO_*x*_–TiO_2_^51^	type II.1	ODHP	wetness impregnation	Precise control of the V loading enables the formation of isolated VO_4_ species which are highly selective for propylene production.	The dependence of VO_*x*_ species on catalyst loading strongly restricts the amount of active metal that can be deposited onto the support and thereby limits the OSC and productivity of the redox catalyst.
(Mo/V)O_*x*_^52^	type II.1	ODHP	coimpregnation	The increased binding strength of V–O following the introduction of Mo into the lattice limits the overoxidation of propane/propylene to CO_*x*_.	Coking may occur at operation temperatures higher than 350 °C. The utilization of V entails ecological risks. The overall OSC is limited due to the inert support material.
(Fe/Mo)O_*x*_^49^	type II.1	ODHP	coimpregnation	Mo doping modulates oxygen evolution from the redox catalyst and increases the surface acidity to achieve an increased propylene yield.	The overall OSC is limited due to the inert support material.
FeVO_4_–VO_*x*_^46^	type II.2	ODHP	wetness impregnation	Hydrogen spillover increases the propylene yield.	The utilization of V entails ecological risks. The overall OSC is limited due to the inert support material.
Ni/HY^48^	type II.2	ODHE	wetness impregnation	The dual active sites allow for individual optimization for the dehydrogenation and hydrogen combustion reaction.	There is comparatively low conversion and therefore low ethylene productivity, which may be attributed to a relatively low OSC. The utilization of Ni entails ecological risks.
La_0.8_Sr_0.2_FeO_3_@Li_2_CO_3_^54^	type II.2	ODHE	wetness impregnation	The molten carbonate layer prevents direct interaction between gaseous hydrocarbons and unselective surface sites of the OC while supplying selective oxygen species to the ODH reaction.	The introduction of a carbonate layer reduces the OSC of the redox catalyst. The molten carbonate layer may lead to difficulties in large scale operation due to reactor corrosion, loss of promoter, and limited possibility of fluidization.
La_0.8_Sr_0.2_FeO_3_@LiBr^84^	type II.2	ODHB	wetness impregnation	The molten LiBr layer prevents direct interaction between gaseous hydrocarbons and unselective surface sites of the OC while creating selective active sites for the ODH reaction.	The introduction of a LiBr layer reduces the OSC of the redox catalyst. The molten alkali halide layer may lead to difficulties in large scale operation due to reactor corrosion, loss of promoter, and limited possibility of fluidization.

## 4 An Outlook on the Industrial Implementation of CL-ODH

### 4.1 Techno-economic Assessments of CL-ODH Processes

Light olefin production via an exothermic ODH process has the potential of energy savings compared to endothermic cracking processes. Moreover, ODH can theoretically achieve higher olefin yields than dehydrogenation, as the product formation is not limited thermodynamically.^85^ The implementation of CL can improve the efficiency of olefin production further by circumventing the necessity of air separation in ODH and facilitate waste heat integration through tailorable exothermic subreactions. To evaluate and quantify the potential benefits of CL-ODH over conventional olefin production processes, several experimental data supported process simulations and techno-economic assessments of CL-ODH systems have been carried out. The following section summarizes selected case studies on different types of CL-ODH systems and their potential energy and CO_2_ emission savings in comparison to established processes for olefin production.

Type I CL-ODH processes have been compared to conventional steam cracking for ethylene production in process simulations. Here, Mg- and Mo-based mixed metal oxides were used as redox catalysts for the selective hydrogen combustion to produce ethylene at 850 °C over up to 1400 h of time on stream (corresponding to 115 redox cycles).^12^ The CL-ODH system reached single pass ethylene yields of up to 68% at over 85% ethane conversion, thus outperforming ethane cracking, which is restricted thermodynamically to an ethylene yield of ∼55% at 70% ethane conversion at temperatures of 750–875 °C.^85^ The simulation predicted a 76% decrease in primary energy consumption of the CL-ODH process compared to conventional steam cracking operating at near-perfect thermal efficiency, which was largely ascribed to the net exothermic ODH reaction.^12^ While the upstream ethylene production process was estimated to bring exergy savings of up to 58%, the downstream product separation of the CL-ODH process can also achieve exergy savings of up to 28% compared to steam cracking. The exergy savings in downstream product separation were largely attributed to the facile separation of water (instead of hydrogen) from the ethylene-containing product stream, which significantly reduces the volume of gas that must be compressed and cooled down for further product separation. According to the process simulation, the combined benefits of the CL-ODH process amount to a reduction in CO_2_ emissions of up to 87% compared to steam cracking.^12^ These results coincide with a further study on Mg- and Mn-based redox catalysts for selective hydrogen combustion in ethylene production via CL-ODH, suggesting a reduction of 82% in energy consumption and a reduction of 82% in CO_2_ emissions compared to ethane cracking.^85^

Analogously, type II.1 CL-ODH processes have been simulated to assess their economic and ecological viability. Chen et al.^53^ modeled a CL-ODH process using a VO_*x*_ on CeO_2_ core–shell redox catalyst at 600 °C. The potential energy savings for propylene production were estimated to be ∼45% compared to the commercially established Oleflex process. Moreover, the simulations predicted a reduction in CO_2_ emissions of ∼40% of the CL-ODH scheme over the Oleflex process. Similar results were obtained in a study evaluating Mn-based oxides as redox catalyst for the CL-ODH-based production of propylene at 450 °C. Owing to the exothermic ODH reaction and utilizing waste heat to preheat the process gas streams, 45% of energy savings were achieved in the simulations compared to the Oleflex process.^86^

Brody et al.^87^ carried out an economic assessment of CL-ODH using Li_2_CO_3_-promoted LSF (type II.2 redox catalyst) for ethylene production. The CL-ODH system was investigated experimentally over 1200 h, corresponding to over 4000 redox cycles, yielding an ethylene selectivity of ∼90% at 67% ethane conversion. Owing to the high reaction temperature chosen (735 °C), gas phase dehydrogenation has likely contributed significantly to the ethylene yield. Consequently, the Li_2_CO_3_-promoted LSF also acted as a redox catalyst for selective hydrogen combustion. Process modeling using the experimentally observed catalytic performance suggested that, despite the endothermic gas phase dehydrogenation of ethane, a net exothermic heat of reaction for autothermal operation is achievable over a wide range of process parameters, if the gas streams are preheated to 350 °C. According to the model, preheating of the reactant gas streams can be realized by heat integration of the regenerator reactor of the CL system in which the redox catalyst is reoxidized. The study evaluated the CL-ODH process in combination with a subsequent oligomerization of ethylene to yield mid-distillate fuels, and the techno-economic analysis predicted a commercially attractive fuel price of under $0.53/L ($2/gal), due to the high ethylene selectivity and overall net exothermic reaction. Similar results were obtained in a techno-economic assessment of a Na_2_MoO_4_-modified CaTi_0.1_N_0.9_O_3_ core–shell redox catalyst in a CL-ODH of ethane based process for liquid fuel production.^55^

Luongo et al.^47^ carried out an experimentally supported techno-economic assessment of a type II.2 CL-ODH process for ethylene production. The process simulation model was based on an Sr_0.8_Ca_0.2_FeO_3_ perovskite that provided gaseous oxygen for the conversion of ethane to ethylene over a VO_*x*_/SiO_2_ catalyst in a subsequent ODH reactor. In this study, up to 28% energy savings compared to the conventional steam cracking process were predicted. Most of the reduction in the energy consumption was due to the exothermic ODH reaction, as well as a high overall ethylene selectivity with fewer byproducts, which minimizes the amount of downstream separation units. A reduction of the number of the necessary separation units also contributed to lower capital costs of the CL-ODH process compared to steam cracking, leading ultimately to a decrease in the costs of ethylene production by 21%.

The key results of selected process simulations and techno-economic assessments for different types of CL-ODH processes to produce olefins are summarized in Table 3. In all of the cases explored, olefin production through CL-ODH compared favorably to established processes such as steam cracking or direct dehydrogenation. Energy savings of up to 87% were achieved, and the main driving forces for the reduced energy consumption and CO_2_ emissions in the CL-based processes were the exothermic ODH reaction compared to the endothermic cracking reaction and the reduction of downstream product separation efforts.

**Table 3:** Summary of Key Performance Indicators of CL-ODH Schemes (Ethane, ODHE; Propane, ODHP) Obtained from Process Simulations^a^

reaction	catalyst type	redox catalyst	energy savings (%)	CO_2_ savings (%)	product price
ODHE	type I	MnO_*x*_–MgO^85^	82	82	n.a.
ODHE	type II.2	La_0.8_Sr_0.2_FeO_3_–Li_2_CO_3_^87^	n.a.	n.a.	$0.5/L ($1.88/gal)^b^
ODHE	type I	CaTi_0.1_Mn_0.9_O_3_–Na_2_MoO_4_^55^	n.a.	n.a.	$0.41/L ($1.57/gal)^b^
ODHE	type I	Mg_6_MnO_8_^12^	76	87	n.a.
ODHE	type II.2	Sr_0.8_Ca_0.2_FeO_3_ + V_2_O_5_–SiO_2_^47^	28	24	€460/ton ($0.49/kg)
ODHP	type II.1	VO_*x*_–CeO_2_^53^	45	40	n.a.
ODHP	type II.1	Mn_2_O_3_^86^	45	n.a	n.a.

a Energy and CO_2_ savings were calculated with respect to established ethylene (steam cracking) and propylene (Oleflex) production processes.

b The final product was converted to liquid fuels (C_4_–C_10_) in an oligomerization unit using ethylene as a reaction intermediate.

### 4.2 Implementation of Redox Catalysts in Fluidized Bed Reactors

To implement a CL scheme in an industrial process efficiently, fluidized bed reactors are generally proposed to facilitate the transportation of the redox catalyst between the reducer and regenerator/oxidizer.^88^ In most academic studies however, fixed bed reactors are used to assess the performance of the redox catalyst for CL. The fluidization of the redox catalyst may in fact greatly compromise the lifetime of the material, due to particle breakage, abrasion, and attrition caused by mechanical stress. This might also lead to the deterioration of their surface modification, which is paramount to maintain a high olefin selectivity in particular when molten carbonate or molten salt layers are employed.

One of the first studies on fluidized redox catalysts for CL-ODH was carried out by Al-Gahmdi et al.,^88^ investigating a VO_*x*_/γ-Al_2_O_3_ type II.1 redox catalyst for the CL-ODH of ethane in a fluidized bed riser simulator. Approximately 58% ethylene selectivity at 28% ethane conversion was achieved at 600 °C. The best catalytic performance was attained by injecting multiple ethane pulses into the fluidized bed before regeneration, as opposed to regenerating the redox catalyst in air after each ethane pulse. The rising ethylene yield with increasing number of ethane pulses was ascribed to a higher ethylene selectivity of the partially reduced redox catalyst. It was proposed that this mode of operation could be realized in an industrial process comprised of a twin circulating fluidized bed setup, in which the majority of the redox catalyst is recirculated in the reducer, and only a fraction of the particles is transferred to the regenerator. Similar studies have been carried out for the CL-ODH of propane, reporting 85% propylene selectivity at 28% propane conversion when using VO_*x*_/ZrO_2_-γ-Al_2_O_3_ as the redox catalyst at 550 °C.^89,90^

While these studies demonstrate the general feasibility of carrying out CL-ODH reactions in a fluidized bed reactor, the long-term implications of such a mode of operation (e.g., deactivation of the redox catalyst due to mechanical wear) were not addressed. Neal et al.^12^ investigated an Mg_6_MnO_8_-based redox catalyst for ethane CL-ODH in a fluidized bed reactor and demonstrated its stability over 1400 redox cycles. Over 10 days of continuous operation, the ethane conversion increased from ∼83 to 87%, while the ethylene selectivity dropped only slightly from ∼90 to 88%. These results paint a promising picture for the implementation of fluidized bed reactors in CL-ODH, but the long-term stabilities of redox catalysts that contain complex surface modifications such as molten salts remain unexplored under fluidized conditions.

## 5 Challenges and Perspectives

This review has presented recent developments in (redox) catalyst engineering, which have surpassed the catalytic performances of existing ODH catalysts. Although the presented redox catalysts have been reported to achieve high olefin selectivity and alkane conversion, there remains a large research gap from the development of redox catalysts at the lab scale to their industrial implementation.

First, capital expenses should be considered in the implementation of an economically viable CL-ODH scheme. The cost of raw materials should guide the design of industrial redox catalysts, and the price of rarer elements such as V currently significantly exceeds that of Fe or Al.^92^ Furthermore, the complexity in the redox catalyst synthesis contributes significantly to the overall cost of the CL scheme. For instance, the addition of surface modifiers such as alkali salts or a carbonate layer, which are required to cover the substrate uniformly, complicates the scalability of these materials and increases their production cost.

Second, the operating expenses of the CL-ODH process need to be minimized. The redox catalysts should aim at achieving a high OSC, which would translate into a high throughput, allowing minimization of the need for purge steps between the reduction and regeneration steps of the redox catalyst. Currently, the OSC of many reported redox catalysts (in particular those for which the active metal oxide catalyst, e.g., VO_*x*_, is supported on inert structural stabilizers such as Al_2_O_3_ or TiO_2_) in CL-ODH schemes appears too low to find application in an industrial context.^26^ Further, a high mechanical strength and stability are essential for long-term performance. Currently, the cyclic stabilities of redox catalysts are often reported to be of the order of 100 redox cycles, which is insufficient at the industrial scale for which redox catalysts are required to display stable catalytic performances of the order of several 1000 redox cycles. In particular, the use of molten salts as coatings may lead to a gradual performance loss over redox cycling, although it has been proposed that careful process engineering may circumvent this problem, e.g., by bleeding LiBr into the reactor for replenishment.^84^ The environmental impact of materials should also be considered. For example, Ni, Cr, and V are known to be harmful to biodiversity, contaminating water reservoirs and soil, and their use should, therefore, be minimized or ideally avoided.^83,91,92^ At the industrial level, the disposal of toxic and nonrecyclable materials greatly increases the operating costs, and further research into regeneration techniques of spent catalysts is needed.

The presented process simulations and techno-economic studies suggest that CL-ODH may achieve extensive savings in energy consumption and CO_2_ emissions of more than 80% over conventional olefin production processes, mainly due to the exothermic ODH reaction and simplified downstream processing. While some of the studies presented here based their process simulations on experimental data obtained in a laboratory, it is essential to validate the redox catalyst on a pilot-plant scale, to properly examine the feasibility of implementing CL-ODH on an industrial level. In this context, it is also paramount to evaluate critically the process and reactor design with respect to the CL-ODH redox catalyst. Some redox catalysts, e.g., containing molten salt surface modifications, may not be applicable within conventional fluidized reactor setups due to a shortened catalyst lifetime caused by the enhanced mechanical wear under fluidized conditions. Alternative reactor types, such as moving bed reactors, should therefore also be investigated to alleviate possible material restrictions of the redox catalyst enforced from a process standpoint. Moreover, accounting for potential impurities in the feedstock or fluctuating reactor outputs should be considered in process simulations, as materials can experience a large variability in performance when deviating from the established operating point. Thus, further research is required to bridge the gap to the industrial implementation of the presented redox catalysts.

While significant progress in the development of redox catalysts for CL-ODH has been made recently, further advancements in olefin selectivity and overall activity would benefit the potential of its industrial implementation. We therefore suggest applying the presented characterization techniques for identifying nucleophilic and electrophilic oxygen species to promising CL-ODH redox catalysts to attain a better understanding of material properties that favor a higher selectivity toward ODH. Discovering such structure–performance relationships may then be utilized to establish rational guidelines for CL-ODH redox catalyst design. Furthermore, the increasing penetration of artificial intelligence and machine learning (ML) approaches in the chemical and engineering sciences has sparked the implementation of novel approaches for highly efficient material screenings. In CL, ML-based screening methods have already been developed and applied to propose material compositions for OCs and redox catalysts in the fields of CL combustion, CL air separation, and CL oxidative coupling of methane.^93−96^ The ML-based selection of catalyst materials may also be coupled with a fully automated, high-throughput synthesis to feed the ML model with experimental data for a robust and efficient material screening. This approach has already been carried out utilizing common synthesis methods such as impregnation, a preparation method that was employed to fabricate a majority of the CL-ODH redox catalysts presented in this review.^97^ Hence, ML-guided material screening procedures pose a great potential to identify novel and improved material compositions for CL-ODH redox catalysts.

## 6 Conclusion

CL-ODH has the potential to reduce the energy consumption, CO_2_ emissions, and complexity of downstream processing of olefin production compared to established technologies and processes. Despite its large potential, CL-ODH has not reached commercial implementation due to the tendency of redox catalysts to overoxidize alkanes to CO_*x*_, thus diminishing the olefin yield and process efficiency. A key factor in increasing the olefin selectivity of redox catalysts for CL-ODH is therefore to understand the involvement of selective (nucleophilic) and unselective (electrophilic) oxygen species in ODH reactions and developing material engineering solutions to control their evolution under different reaction conditions. Characterization techniques such as XPS, Raman spectroscopy, RIXS, and EPR spectroscopy have been employed to detect electrophilic and nucleophilic oxygen species in metal oxides. Further, different material engineering solutions have been developed to tune the reactivity of oxygen species in redox catalysts. Regarding redox catalysts for the selective combustion of hydrogen (type I), the introduction of dopants and surface layers was shown to reduce the number of sites that favor CO_2_ production, thus substantially increasing hydrogen combustion selectivity. In the case of bifunctional redox catalysts (type II.1), doping the metal oxide with cations of higher oxidation state than the host lattice was shown by hydrogen- and propane-TPR to strengthen the M–O bond and thereby reduce overoxidation and improve olefin selectivity. In the case of tandem material systems with a split functionality (type II.2), core–shell-type structures consisting of catalytically active coatings that block the nonselective sites of the redox catalyst displayed outstanding abilities in increasing the olefin yield. Another successful strategy to increase the olefin yield of CL-ODH was to combine materials which individually catalyze the dehydrogenation and selective hydrogen combustion and reduce the distance between their respective active sites to the nanoscale. As such, the oxygen reactivity of each material could be tailored individually toward the desired reaction, while a nanoscale proximity can enhance the interplay of the subreactions. Based on these promising redox catalyst architectures, process simulations and techno-economic assessments have been carried out to prove the economic and ecological viability of CL-ODH, finding energy and CO_2_ emission savings of up to 80% compared to established olefin production processes such as steam cracking. While these results are encouraging, CL-ODH needs to be evaluated at a larger scale and under practically relevant operating conditions to demonstrate its practical feasibility.

## Abbreviations

AIMD: ab initio molecular dynamics
CL: chemical looping
DRIFTS: diffuse reflectance infrared Fourier transform spectroscopy
DSC: differential scanning calorimetry
EDS: energy-dispersive spectroscopy
EIS: electrochemical impedance spectroscopy
EPR: electron paramagnetic resonance
EXAFS: extended X-ray absorption fine structure
GHSV: gas hourly space velocity
HAADF: high angle annular dark field
LEIS: low-energy ion scattering
LSF: La_0.8_Sr_0.2_FeO_3_
ML: machine learning
MvK: Mars–van Krevelen
MS: mass spectrometry
NMR: nuclear magnetic resonance
NPD: neutron powder diffraction
ODH: oxidative dehydrogenation
OSC: oxygen storage capacity
RIXS: resonant inelastic X-ray scattering
STEM: scanning transmission electron microscopy
TM: transition metal
TPD: temperature programmed desorption
TPR: temperature programmed reduction
XANES: X-ray adsorption near edge spectroscopy
XAS: X-ray absorption spectroscopy
XPS: X-ray photoelectron spectroscopy
XRD: X-ray diffraction
